# Epidemiology of mutant *Plasmodium falciparum* parasites lacking histidine-rich protein 2/3 genes in Eritrea 2 years after switching from HRP2-based RDTs

**DOI:** 10.1038/s41598-021-00714-8

**Published:** 2021-10-26

**Authors:** Selam Mihreteab, Karen Anderson, Cielo Pasay, David Smith, Michelle L. Gatton, Jane Cunningham, Araia Berhane, Qin Cheng

**Affiliations:** 1 National Malaria Control Program, Ministry of Health, Asmara, Eritrea; 2 The Australian Defence Force Malaria and Infectious Disease Institute Laboratory, QIMR Berghofer Medical Research Institute, Brisbane, Australia; 3 Drug Resistance and Diagnostics, Australian Defence Force Malaria and Infectious Disease Institute, Brisbane, Australia; 4 School of Public Health and Social Work, Queensland University of Technology, Brisbane, Australia; 5 Global Malaria Programme, World Health Organization, Geneva, Switzerland; 6 Communicable Diseases Control, Ministry of Health, Asmara, Eritrea

**Keywords:** Genetics, Microbiology, Molecular biology, Health care, Medical research

## Abstract

Eritrea was the first African country to complete a nationwide switch in 2016 away from HRP2-based RDTs due to high rates of false-negative RDT results caused by *Plasmodium falciparum* parasites lacking *hrp2/hrp3* genes. A cross-sectional survey was conducted during 2019 enrolling symptomatic malaria patients from nine health facilities across three zones consecutively to investigate the epidemiology of *P. falciparum* lacking *hrp2/3* after the RDT switch. Molecular analyses of 715 samples revealed the overall prevalence of *hrp2-, hrp3*-, and dual *hrp2/3*-deleted parasites as 9.4% (95%CI 7.4–11.7%), 41.7% (95% CI 38.1–45.3%) and 7.6% (95% CI 5.8–9.7%), respectively. The prevalence of *hrp2-* and *hrp3-*deletion is heterogeneous within and between zones: highest in Anseba (27.1% and 57.9%), followed by Gash Barka (6.4% and 37.9%) and Debub zone (5.2% and 43.8%). *hrp2/3*-deleted parasites have multiple diverse haplotypes, with many shared or connected among parasites of different *hrp2/3* status, indicating mutant parasites have likely evolved from multiple and local parasite genetic backgrounds. The findings show although prevalence of *hrp2/3*-deleted parasites is lower 2 years after RDT switching, HRP2-based RDTs remain unsuitable for malaria diagnosis in Eritrea. Continued surveillance of *hrp2/3*-deleted parasites in Eritrea and neighbouring countries is required to monitor the trend.

## Introduction

Malaria has been a major public health problem in Eritrea, with 70% of the population living in malaria endemic areas^1^. In 2006 the National Malaria Control Programme (NMCP) of Eritrea began implementing malaria rapid diagnostic tests (RDTs) as part of malaria control strategy, initially at health station level and later to Community Health Agents. A combination RDT was selected to allow diagnosis of both *Plasmodium falciparum* and *P. vivax* malaria and has been the mainstay diagnostic tool for malaria in the country. In 2015, the NMCP conducted an investigation into reports of false negative results with SD Bioline Pf/Pv RDT which targets histidine rich protein 2 (HRP2) for diagnosing *P. falciparum* and *P. vivax* specific plasmodium lactate dehydrogenase (Pv-LDH) for diagnosing *P. vivax*^2^. The investigation did not reveal any RDT defects nor operator errors associated with false negative RDT results. This led to a strong suspicion for *hrp2/3* deletions being the major cause of the false negative RDTs as many *P. falciparum* samples were positive by microscopy and positive on the pan-pLDH line of different RDT products. A further survey was conducted at two hospitals in the Northern Red Sea zone in February–March 2016, with molecular analysis revealing a high prevalence of mutant parasites having dual *hrp2/3*-deletions (Ghindae Hospital: 81%; Masawa Hospital: 42%)^3^. This placed Eritrea amongst the countries with the highest reported prevalence of *hrp2/3*-deletions globally^4^. Based on these findings, the NMCP withdrew HRP2-based RDTs from the country in mid-2016 and reverted to presumptive treatment of malaria for 6 months in places where microscopy was not available until an alternative RDT could be procured and implemented.

**Figure 1:**
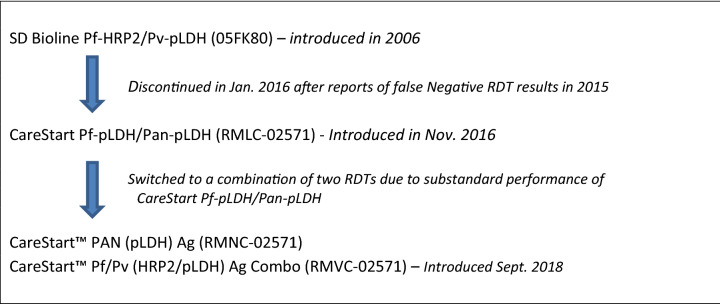
Schematic illustration of the introduction and switching of malaria RDTs in Eritrea.

Eritrea was the first country in Africa to undertake a nationwide switch of RDTs, and it has been a challenging process due to the lack of commercially available and WHO prequalified non-HRP2 based combination RDTs with proven performance against *hrp2/3*-deleted parasites. Although a single alternative Pf-pLDH and pan-pLDH RDT was initially used to replace HRP2-based RDT in 2016, the final solution implemented in 2018 consists of a two-step diagnostic algorithm where a pan-pLDH only RDT is used, followed by a Pv-specific combination test for all patients who test positive to the pan-LDH test (Fig. 1). Significant retraining of health workers was required to implement the new RDTs and diagnostic algorithm.

The nationwide change in RDT type in Eritrea provides a unique study site and population to evaluate the impact of removing HRP2-based RDT selection pressure on the epidemiology of *hrp2/3-*deleted *P. falciparum* parasites. To this end, a cross-sectional survey was conducted at multiple health facilities in Eritrea in 2019, with the primary aim of re-assessing the prevalence of parasites lacking *hrp2/3* genes 2 years after removal of HRP2-based RDTs for case management. The secondary objective was to compare parasite population genetics between the 2016 and 2019 surveys.

## Results

### Study sites and sample collection

**Figure 2:**
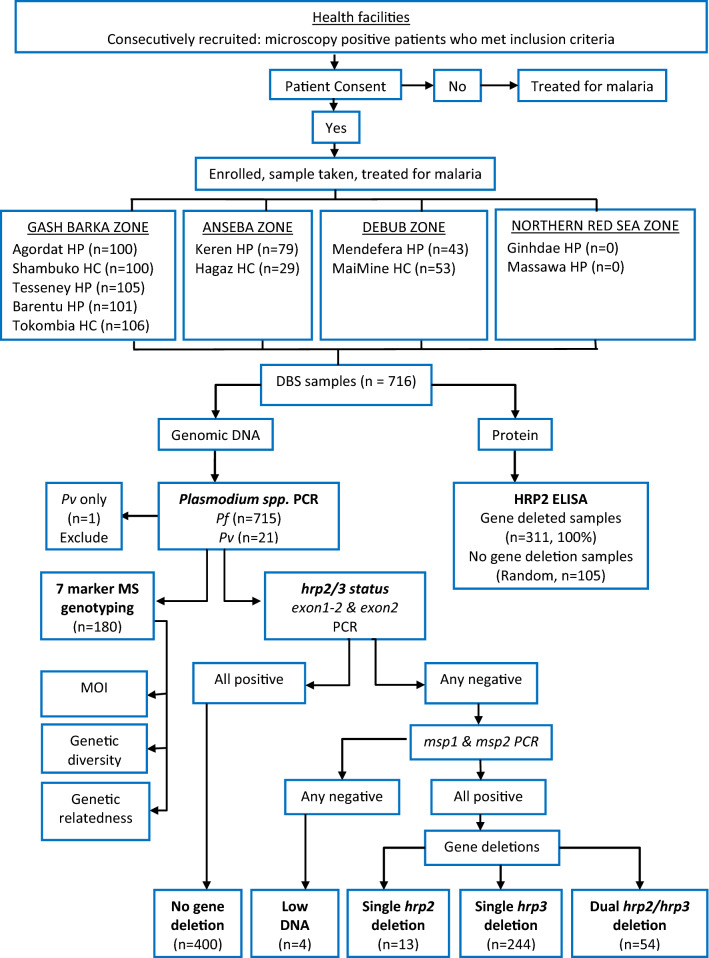
Patient recruitment, blood sample collection and laboratory analyses workflow.

A cross-sectional survey was conducted across 11 health facilities located in four zones of Eritrea, through the 2019–2020 transmission season (January 2019 to January 2020). These zones were selected because of previously reported high rates of false negative RDTs or *pfhrp2/3* deletions^2,3^. All selected health facilities have microscopy services implemented with a quality assurance system including regular refresher training and competency assessment of microscopists. A total of 716 dried blood spot (DBS) samples were collected consecutively from patients with fever within the previous 48 h, with microscopy-confirmed *P. falciparum*. No samples were collected from Ghindea and Massawa Hospitals in the Northern Red Sea zone, thus all 716 collected DBS originated from nine health facilities in three zones (Table 1). Patients enrolled in the study were 69% (494/716) males and 31% (222/716) females, with 99.2% symptomatic at time of enrolment. Patient age ranged from 1 to 85 years, with a mean age of 26.7 years (95% CI 25.4, 28.0) (Table 1). Patient enrolment, sample collection and laboratory analyses procedures are outlined in Fig. 2.

**Table 1:** Source of samples, period of collection, patient demographics and PCR results.

Zone	Health facility*	Collection period	n	Age		Gender		Symptomatic (%)	PCR results		
				Range	Mean (95%CI)	Male (%)	Female (%)		Pf only	Pv only	Pf + Pv
GASH BARKA	Agordat HP	Jan–Nov 19	100	3–70	26.8 (23.8–29.7)	75	25	98.0	94	0	6
	Shambuko HC	Feb–Nov 19	100	1–69	19.6 (16.9–22.3)	57	43	100	99	0	1
	Tesseney HP	Feb–Nov 19	105	2–76	28.1 (24.7–31.5)	69	36	98.1	104	0	1
	Tokombia HC	Feb–Nov 19	106	2–80	25.1 (21.3–28.8)	70	36	100	106	0	0
	Barentu HP	Sep–Nov 19	101	1–85	23.5 (20.4–26.7)	68	33	100	101	0	0
ANSEBA	Keren HP	Feb 19–Jan 20	79	8–84	36.4 (32.1–40.6)	66	13	100	68	1	10
	Hagaz HC	Feb 19–Jan 20	29	3–70	35.4 (29.0–41.9)	25	4	96.6	29	0	0
DEBUB	Mendefera HP	Feb 19–Jan 20	43	1–62	31.7 (27.1–36.2)	33	10	97.7	43	0	0
	MaiMine HC	Feb 19–Jan 20	53	4–69	22.2 (17.9–26.4)	31	22	100	51	0	2
NORTHERN RED SEA	Ghindae HP	Feb 19–Jan 20	0	N/A	N/A	N/A	N/A	N/A	N/A	N/A	N/A
	Massawa HP	Feb 19–Jan 20	0	N/A	N/A	N/A	N/A	N/A	N/A	N/A	N/A
Total			716	1–85	26.7 (25.4–28.0)	494	222	99.2	695	1	20

*HP hospital, *HC* Health center.

### *Plasmodium spp* determined by PCR

A multiplex PCR revealed that 715/716 samples were positive for *P. falciparum* and 21/716, positive for *P. vivax*. Combined, 695 samples were infected with *P. falciparum* only, one with *P. vivax* only and 20 with *P. falciparum* plus *P. vivax* (Table 1). *P. vivax* positive samples were detected in DBS collected from five health facilities, with Keren Hospital, Anseba (11/79, 14%) and Agordat Hospital (6/100, 6%) in Gash Barka zone having the highest proportions (Table 1). The single *P. vivax* only sample was excluded from further analysis, while mixed species samples were included in the *hrp2/3* deletion analysis.

### Prevalence of parasites with gene deletion

**Figure 3:**
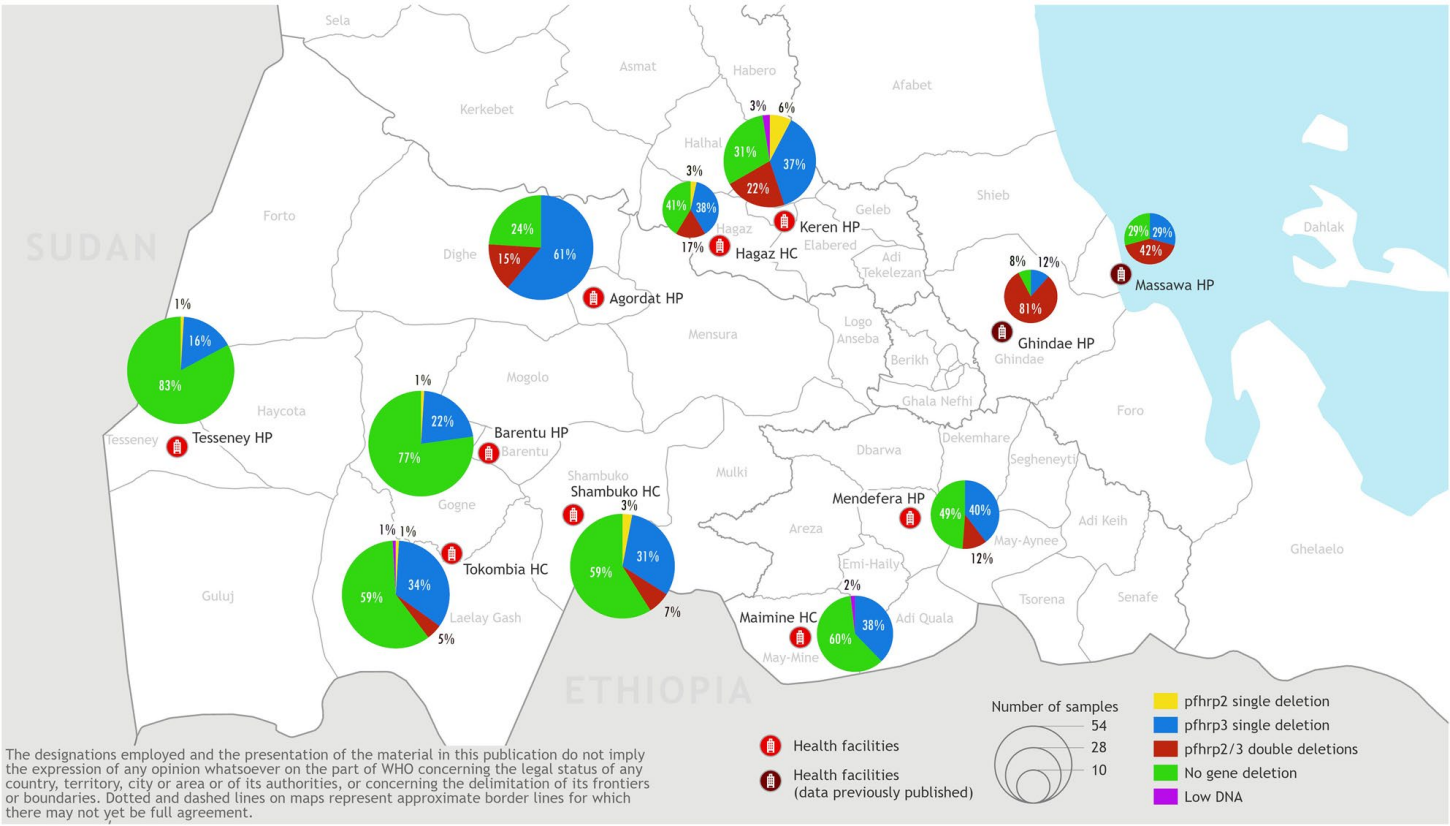
Geographical distribution and proportion of parasites having deleted *hrp2*, *hrp3* and dual *hrp2/3* genes. Map was generated by WHO GIS Centre for Health, Department of Data and Analytics (DNA) /Division of Data Analytics and Delivery for Impact (DDI) using ArcGIS Pro 2.8.2 (https://pro.arcgis.com/en/pro-app/latest/get-started/get-started.htm).

The *hrp2/3* gene status was determined for all 715 *P. falciparum* positive samples. A total of 67 (9.4%, 95% CI 7.4–11.7%) samples were classified as having deleted the *hrp2* gene. Deletions of *hrp3* were identified in 298 (41.7%, 95% CI 38.1–45.3%) samples. No *hrp2* or *hrp3* deletions were observed in 400 samples (55.9%, 95% CI 52.3–59.5%), while four samples (0.6%) gave inconclusive results due to insufficient DNA. Combining the individual gene deletion results showed that 13/715 (1.8%, 95% CI 1.0–3.1%) of samples had only a *hrp2* gene deletion, 244/715 (34.1%, 95% CI 30.7–37.7%) had only a *hrp3* gene deletion and 54/715 (7.6%, 95% CI 5.8–9.7%) had deleted both the *hrp2* and *hrp3* genes (Fig. 3, Supplementary Fig. 1).

### Geographical heterogeneity

Geographical distribution and prevalence of *hrp2* and *hrp3-*deleted parasites are shown in Fig. 3. *hrp2*-deleted parasites were detected in eight of the nine health facilities and within all three of the surveyed zones, with Anseba zone having significantly higher prevalence than the other zones (*P* < 0.001). The overall prevalence of *hrp2* and *hrp3* deletions was 6.4% (33/512; 95% CI 4.6–8.9%) and 37.9% (194/512; 95% CI 33.8–42.2%) respectively for the Gash Barka zone, 27.1% (29/107; 95% CI 19.6–36.3%) and 57.9% (62/107, 95% CI 48.5–66.9%) respectively for the Anseba zone, and 5.2% (5/96, 95% CI 1.9–11.9%) and 43.8% (42/96, 95% CI 34.3–53.7%) for the Debub zone.

Prevalence of *hrp2* deletion ranged from 0% (95% CI 0.0–8.1%) in MaiMine Health Centre (Debub zone) to 29.5% (95% CI 20.5–40.2%) in Keren Hospital (Anseba zone). *hrp3-*deleted parasites were detected in all nine health facilities, with prevalence ranging from 16.2% (95% CI 10.3–24.5%) in Tesseney Hospital to 76% (95% CI 66.7–83.4%) in Agordat Hospital within the Gash Barka zone.

Dual *hrp2/3*-deleted parasites were detected in six of the nine health facilities in the three zones: 3/5 facilities in Gash Barka zone, 2/2 facilities in Anseba zone and 1/2 facilities in the Debub zone. The prevalence of dual *hrp2/3*-deleted parasites at Keren Hospital (21.8%, 95% CI 14.0–32.2%) and Hagaz Health Centre (17.2%, 95% CI 7.1–35.0%), the two facilities with the highest prevalence in this study, were significantly lower than that previously observed in patients from Ghindae Hospital (80.8%, 95% CI 61.7–92.0%; Fishers exact test, *p* < 0.001). Although the prevalence at both sites was also lower than that previously observed in Massawa Hospital (41.7%, 95% CI 24.4–61.2%), the difference failed to reach statistical significance (Fisher’s exact test, *p* > 0.065).

### Pattern of gene deletions

Among 67 *hrp2-*deleted parasite samples, 37.3% (25/67) had deleted both exons of the *hrp2* gene, while 41% (28/67) had deleted exon1 only and 20.9% (14/69) of samples had deleted exon2 only. Among 298 *hrp3-*deleted parasites, 59.4% (177/298) had deleted both exons of *hrp3*, while 18.5% (55/298) deleted exon1 only and 22.1% (66/298) deleted exon2 only.

### HRP2 levels and *hrp2/3* status

**Figure 4:**
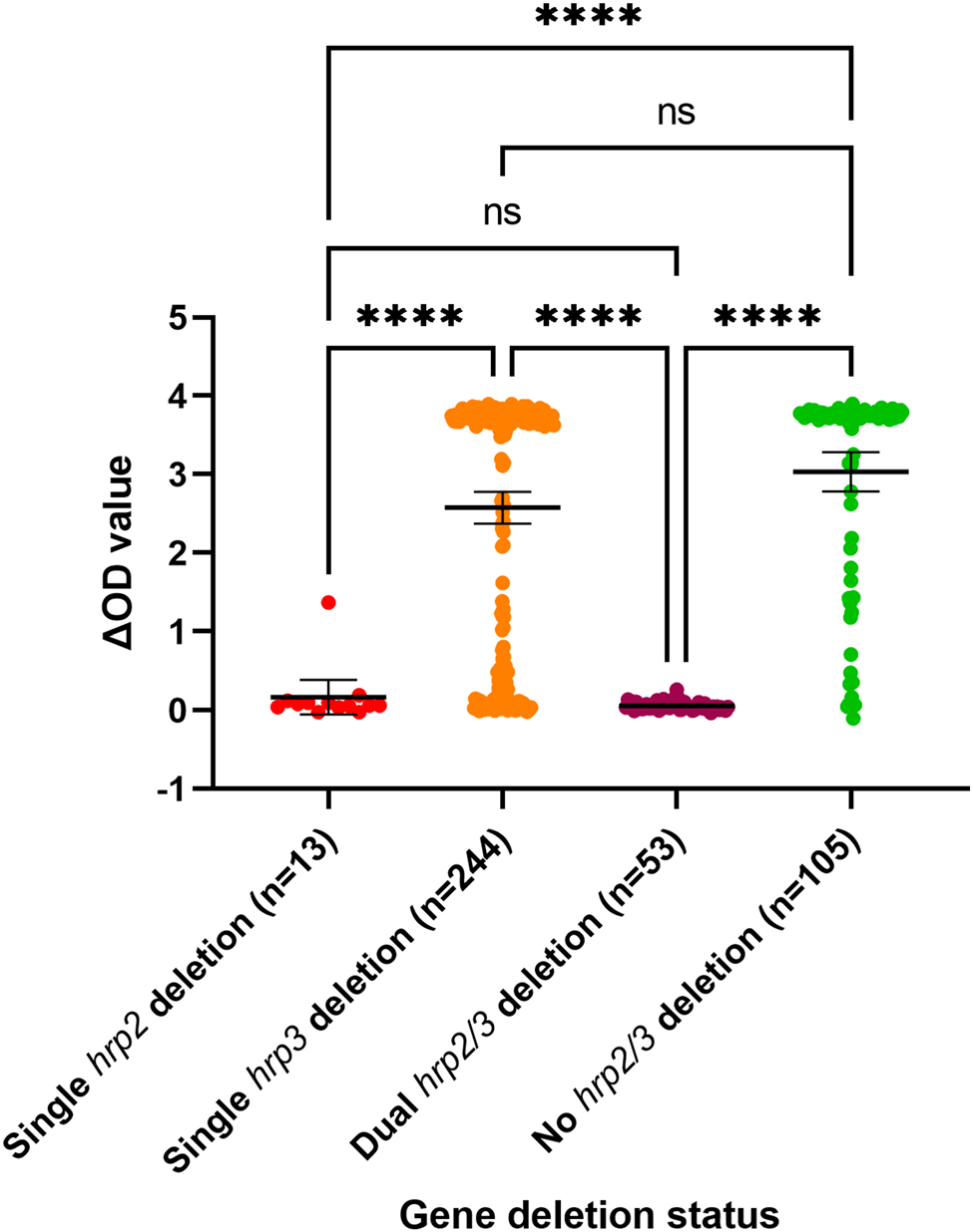
HRP2 level measured by ELISA (*p* values were calculated using Dwass–Critchlow–Fligner pairwise comparisons). ns: not significant; *P < 0.05, ****P < 0.0001.

HRP2 protein levels were measured for all samples classified by PCR as single *hrp2-*, single *hrp3-* and dual *hrp2/3-*deleted parasites, as well as a randomly selected set of samples without gene deletions using ELISA (Fig. 4). The mean ΔOD for 105 samples without *hrp2 and hrp3* deletions was 3.032 (95% CI 2.783–3.282). The mean ΔOD for 244 samples with single *hrp3-*deletions was 2.576 (95% CI 2.373–2.779), not significantly different to samples without gene deletions (*P* = 0.053), but significantly higher than single *hrp2-* or dual *hrp2/3*-deleted parasites (*P* < 0.0001). The mean ΔOD for 13 single *hrp2-*deleted samples was 0.167 (95% CI 0.053–0.388), not significantly different to dual *hrp2/3-*deleted samples (n = 53) which had a mean ΔOD of 0.058 (95%CI 0.043–0.072) (*P* > 0.999). Hence, this ELISA assay detected all samples with dual *hrp2/3* deletions and all single *hrp2* deletions except one sample, and showed little cross reactivity with HRP3. Sequencing revealed that the exceptional parasite had deleted the 3’ end of exon2 but retained exon1 and the 5’ end of the exon 2 of the *hrp2* gene which encoded 37 amino acids (excluding primer sequences) including three type 2 repeats (AHHAHHAADAHHAHHAADAHHAHHAADAHHAAAHH). It is likely that the presence of a small number of type 2 repeats (contains a major epitope recognised by monoclonal antibodies used in malaria RDTs^5^) has contributed to the positive ELISA results although persisting HRP2 from previous infections could not be ruled out.

### Microsatellite typing

180 samples, 20 from each health facility, were randomly selected for genotyping on seven microsatellite markers. The 180 samples included 76.9% (10/13) of the samples classified as single *hrp2*-deletion, 32.8% (80/244) classified as single *hrp3*-deletion, and 52.0% (28/54) of the samples with dual *hrp2/3*-deletions, as well as 15.5% (62/400) of samples without gene deletions (Supplementary Table 1). Genotyping was successful on all seven makers for 173/180 samples.

### Seven-marker haplotypes and haplotype frequency

**Figure 5:**
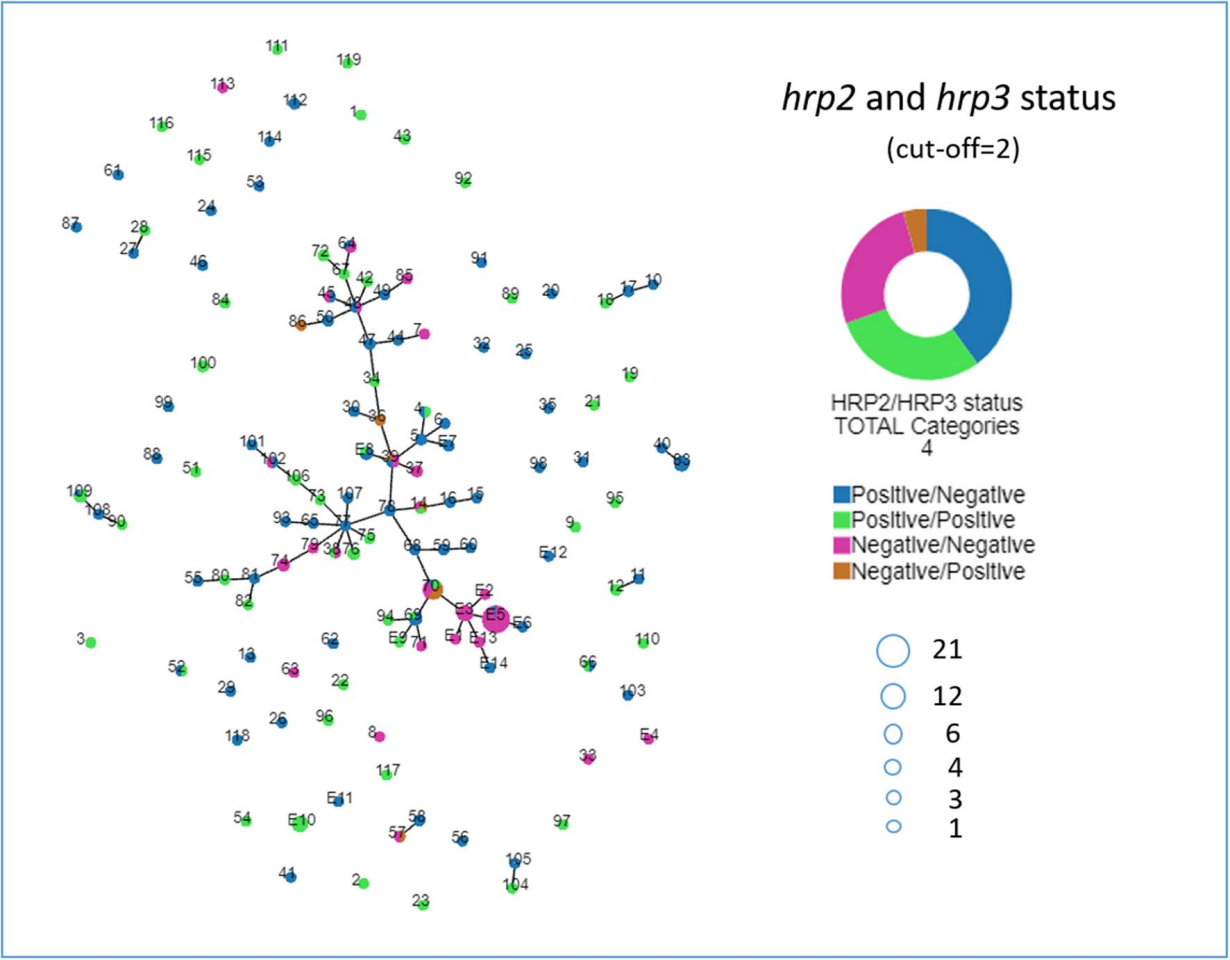
Genetic relatedness of parasite haplotypes (numbered nodes) with varying *hrp2/3* gene status. Parasite haplotypes obtained from our previously published study starts with an E followed with a haplotype number. Parasite haplotypes are connected when ≤ 2/7 markers are different or ≥ 5/7 markers are identical (cut-off = 2).

Genotyping 173 samples revealed 119 unique haplotypes. 89 unique haplotypes were observed in single samples only while the remaining haplotypes were shared by two to 12 samples (Supplementary Fig. 2). Haplotype 70 (shared by 12 different samples) consisted of parasites with 4 different *hrp2/3* gene status. Haplotypes 14 and 39 contained 3, while haplotypes 4, 38, 45, 48, 52, 57, 64, 66, 69, 102 and 109 contained 2 different types of parasites (Fig. 5).

### Genetic diversity of parasites (H_***E***_)

**Figure 6:**
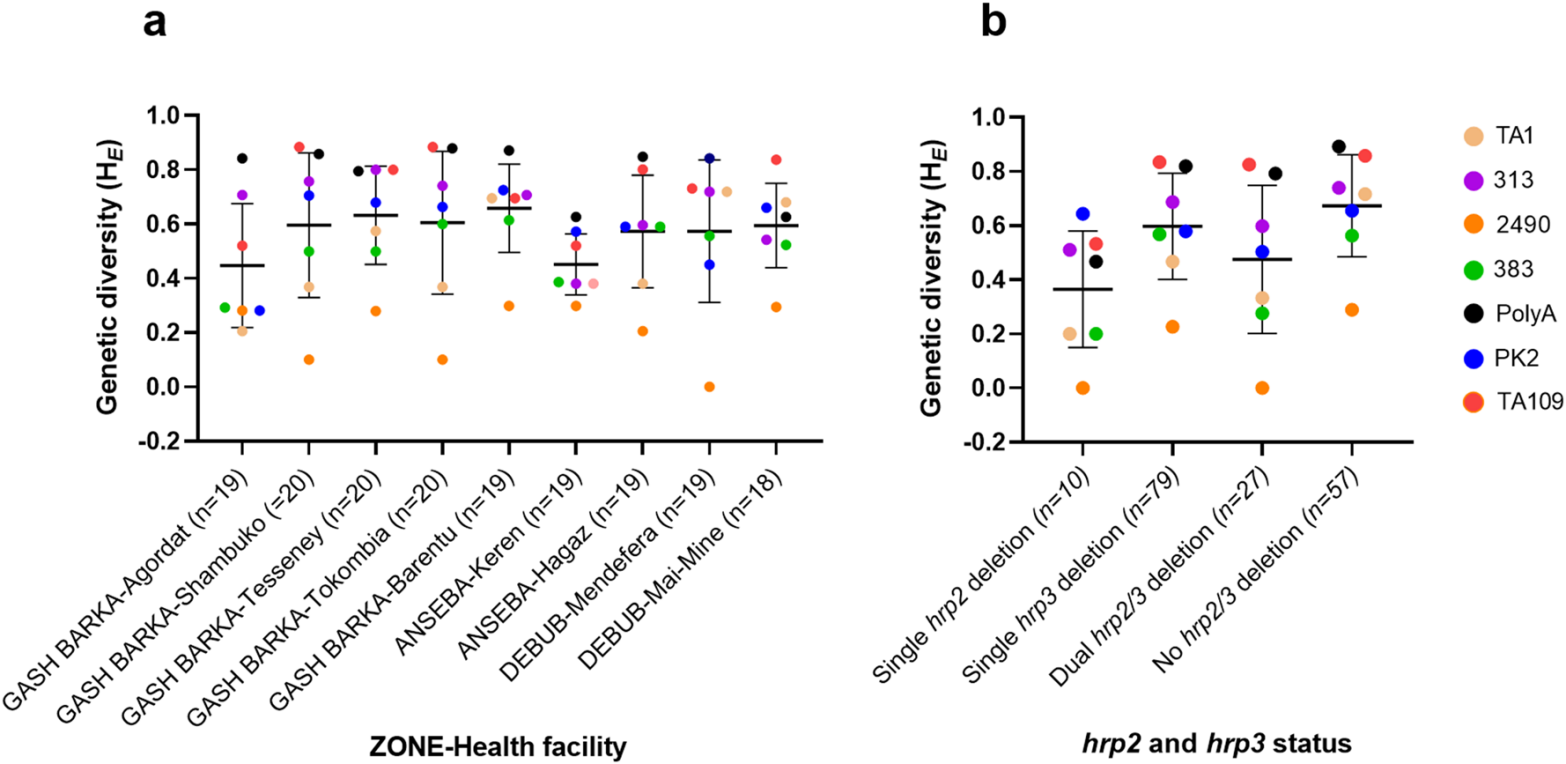
Genetic diversity at seven microsatellite markers in parasites collected from different health facilities (**a**) and with different gene deletion status (**b**). Mean and 95% confidence intervals are shown as lines and error bars, and n represents number of haplotypes.

Genetic diversity of parasite populations at seven markers is shown in Fig. 6. For most health facilities, the PolyA and TA109 marker produced highest H_*E*_ values while 2490, the lowest H_*E*_ values (Fig. 6a). It appeared that parasites from Keren Hospital had lower H_*E*_ values for the majority of markers, while parasites from Barentu Hospital showed consistently high H_*E*_ values for the majority of markers, no significant difference was observed (*P* > 0.05). Level of genetic diversity among parasites of different gene deletion status was not significant different (*P* > 0.05) in all genotyped samples (Fig. 6b).

### Genetic relatedness of parasites

Genetic relatedness of parasites with different *hrp2/3* status is analysed by connecting parasites sharing identical alleles on at least five of the seven microsatellite markers using Phyloviz. A major cluster was revealed connecting 59 parasite haplotypes: 49 obtained from this study and 10 obtained previously from Ghindae and Massawa hospitals in the Northern Red Sea zone (Fig. 5). 14/19 (73.7%) of haplotypes of dual *hrp2/3* deletions, 5/6 (83.3%) of single *hrp2* deletion, and 28/64 (43.8%) of single *hrp3* deletions, as well as 28/48 (58.3%) of intact *hrp2/3* genes from the current study were connected in this major cluster. This indicates that parasites with different gene deletion status are genetically related among themselves, and to parasites without gene deletions.

Unlike previous observations from Ghindae and Massawa hospital where all (E1, E2, E3, E5, E13) but one (E4) haplotype with dual *hrp2/3* deletions were connected, 5/19 haplotypes with dual *hrp2/3* deletions (8, 33, 57, 63 and 113) were not connected to the major cluster. One haplotype with single *hrp2* deletion and 36 haplotypes with single *hrp3* deletions were also outside of the major cluster. Combined, these results demonstrate that *hrp2-* and *hrp3*-deleted parasites in Eritrea have evolved from local parasite populations and from parasites of different genetic back grounds.

**Figure 7:**
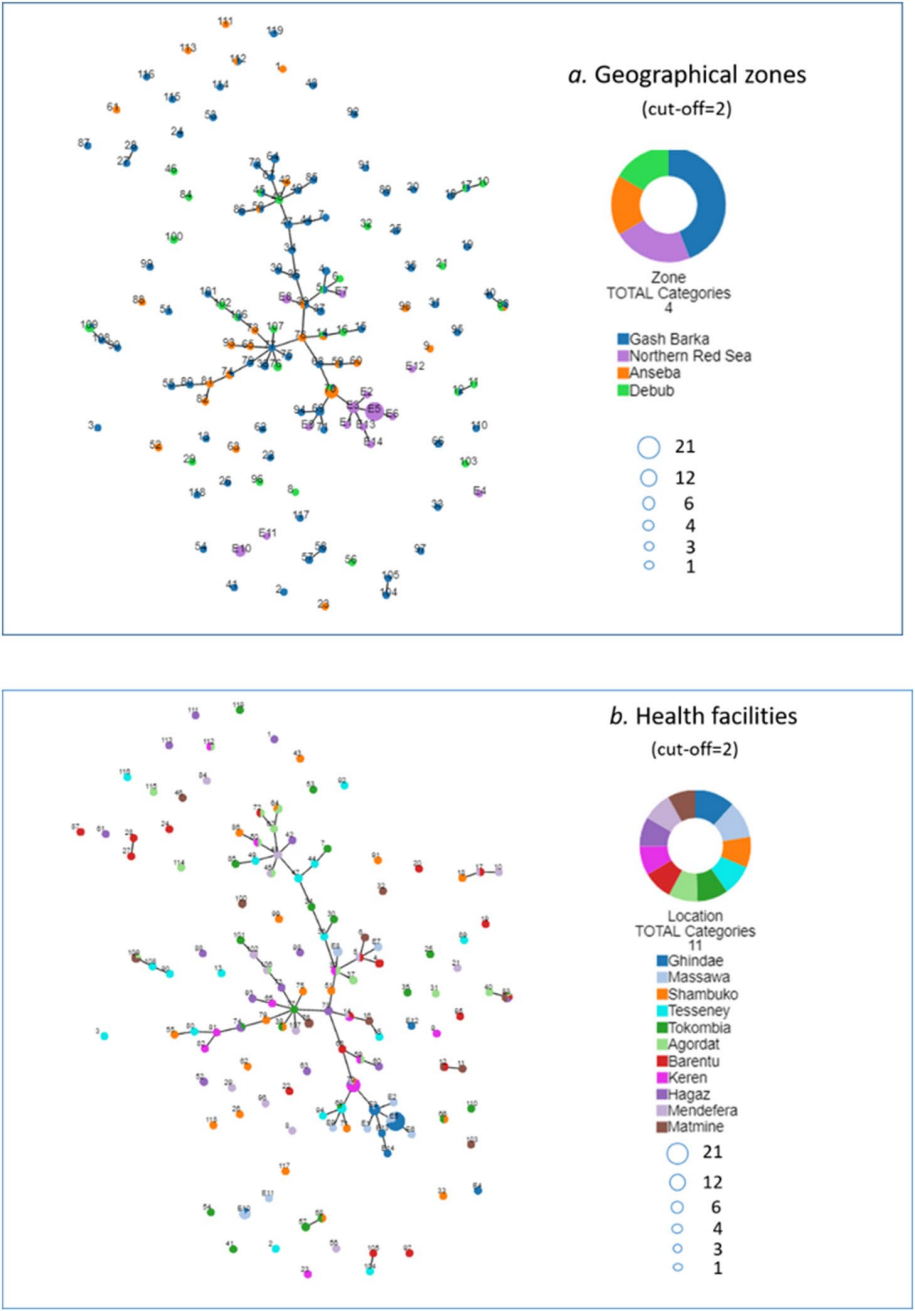
Genetic relatedness of parasite haplotypes (numbered nodes) from different geographical zones (**a**) and from different health facilities (**b**). Parasite haplotypes obtained from our previously published study starts with an E followed with a haplotype number. Parasite haplotypes are connected when ≤ 2/7 markers are different or ≥ 5/7 markers are identical (cut-off = 2).

While parasites from the Northern Red Sea zone tend to cluster on one branch (Fig. 7a), there is no major clustering of parasites between other zones indicating no geographical restrictions on parasite movement. There is no major clustering of parasites within or between different health facilities although several parasite haplotypes from Ghindae Hospital appear to be closely related to some from Keren Hospital (Fig. 7b), possibly indicating frequent human movement between these two areas. The overall lack of parasite clustering observed in different zones may reflect frequent human movement between zones for purpose of trading, schooling and other business, particularly among the youth.

## Discussion

Eritrea was the first African country to switch away from HRP2-based RDTs nationwide due to high rates of false negative RDT results caused by *hrp2/3*-deleted parasites. It is important for the global response strategy to assess the impact of removing HRP2-based RDTs for case management on prevalence trends and epidemiology of *hrp2/3* deletions. This study shows the overall prevalence of *hrp2*, *hrp3* and dual *hrp2/3* gene deletions estimated across three zones in Eritrea is 9.4% (95% CI 7.4–11.7%), 41.7% (95% CI 38.1–45.3%) and 7. 6% (95% CI 5.8–9.7%) respectively. Because HRP2-based RDTs are no longer used in Eritrea it is not possible to describe the prevalence of *hrp2/3* deletions causing false negative RDT results; however, a conservative estimate can be made based on the prevalence of dual *hrp2/3* deletions, which exceeds the WHO 5% prevalence criterion to switch away from HRP2-based RDTs for case management^6^. Therefore, non-HRP2 based RDTs or microscopy remain the most suitable point-of-care malaria diagnostic tools for Eritrea. Currently, malaria diagnosis in Eritrea involves two steps: a pan-pLDH RDT for malaria suspects, and if positive, a Pf (HRP2)/Pv-pLDH RDT is followed to distinguish *P. falciparum* from *P. vivax.*

Prevalence of gene deletions is highly heterogeneous within and between zones. In general, North-west areas of the country have a higher prevalence of *hrp2/3* deletions than South, South-West areas. Keren hospital in the Anseba zone had the highest prevalence of samples containing *hrp2*-deleted (29.5%) and dual *hrp2/3*-deleted (21.8%) parasites, followed by Hagaz Health Centre from the same zone, and then by Agordat Hospital from the neighbouring Gash Barka zone. MaiMine Health Centre in the Debub zone had the lowest *hrp2/3* deletion prevalence, followed by Barentu Hospital in the Gash Barka zone. Prevalence of *hrp2/3*-deleted parasites at any health facilities surveyed in this study was markedly lower than that observed in patients from Ghindae Hospital (81%) in the Northern Red Sea zone in the 2016 survey, while health facilities in the Gash Barka and Debub zones also had lower prevalence than that previously reported for Massawa Hospital (42%)^3^.

Ghindae and Massawa hospitals in the Northern Red Sea zone were included in this survey to enable a direct comparison of *hrp2/3*-deletion prevalence estimates before and 2 years after switching RDTs. However, low patient numbers in these hospitals during the current survey resulted in no samples collected, and prevented such a direct comparison. Nevertheless, the prevalence of *hrp2*-deleted parasites determined in the current survey for the Gash Barka (6.4%), Anseba (27.1%) and Debub zone (5.2%) was much lower than the false negative RDT rate reported for these zones in 2016, 64.7%, 92.9% and 71.4% respectively^2^, although the sample number was relatively small and gene deletion status was not confirmed by molecular analysis in the 2016 RDT study. The prevalence estimates of *hrp2* and *hrp3* deletions determined for Gash Barka zone in the current survey, 6.4% and 37.9%, was also lower than the corresponding prevalence of 10.8% and 47.7% reported for the same zone in a set of samples collected in 2013–2014^7^. Furthermore, the current prevalence estimates of *hrp2*-deleted parasites in Eritrea (9.4%) appears to be lower than that recently reported from areas in neighbouring countries of Ethiopia (50%, 100%)^8,9^, Djibouti (86.5%)^10^, and amongst samples tested in returning travellers from Sudan (11.2%) and South Sudan (17.7%)^11^. This reduction in prevalence may reflect a genuine downward trend following the removal of HRP2-based RDTs for case management in Eritrea, but may also result from different sampling, different levels of genetic diversity and different laboratory methods used. Importantly, data generated from the current survey will provide baseline information for future surveys to be conducted in these areas to determine the trend of gene deletion prevalence.

In this study we performed ELISA to confirm the HRP protein expression status. The ELISA results showed that parasites with dual *hrp2/3* deletions or single *hrp2* deletions had negligible levels of HRP antigens and are expected to be undetectable by HRP2-based RDTs, thus confirming gene deletion status determined by PCRs. While the number of single *hrp2*-deleted parasites was small (n = 13), it was expected that most of these parasites would have produced positive ELISA results due to cross reactivity between HRP3 and anti-HRP2 antibody used in the ELISA kit. However, all but one sample had negligible levels of HRP. This likely resulted from little cross reactivity of antibodies against HRP3 in the ELISA kit, not from insufficient quantity of HRP3 due to low parasitemia since all patients were enrolled based on positive microscopy results.

In contrast to single *hrp2*-deleted parasites, single *hrp3-*deleted parasites showed high levels of HRP and are expected to be detected by HRP2-based RDTs, albeit possibly with slightly lower detection sensitivity than parasites without gene deletions. Therefore, unlike *hrp2*-deleted parasites^12^, single *hrp3*-deleted parasites are not expected to have a selection advantage exerted by use of HRP2-based RDTs. The driving force behind the high prevalence of *hrp3*-deleted parasites in Eritrea is unclear.

In addition to determining gene deletion prevalence, the survey provided a unique opportunity to compare parasite genetic diversity and genetic relatedness between the two surveys. While these analyses were limited to only the dominant haplotypes present in a subset of samples from this study, our data provided strong evidence that that *hrp2/3*-deleted parasites have likely emerged de novo from multiple genetic backgrounds. Firstly, parasites with different *hrp2/3* gene status shared identical haplotypes or showed close genetic connectivity, suggesting they have evolved from same genetic backgrounds; secondly, dual *hrp2/hrp3*-deleted parasites from the current survey have a markedly higher heterogeneity (H_*E*_ = 0.48) than those from the 2016 survey (H_*E*_ = 0.11), and processed a wider number of haplotypes (19 haplotypes in 27 parasite isolates) than those from the 2016 survey (6 haplotypes in 31 parasite isolates) suggesting they have evolved from multiple genetic backgrounds. This implies that besides surveillance and switching RDTs, control as opposed to containment strategies may be most effective for combatting these mutant parasites.

Interestingly, the patterns of *hrp2/3* gene deletion are also much more diverse in the current survey compared to the 2016 survey. In the current survey, we observed parasites deleting *hrp2* only, *hrp3* only and dual *hrp2/3* gene, while in the 2016 survey only dual *hrp2/3*-deleted and single *hrp3-*deleted parasites were observed. In addition, we observed significant proportions of parasites having deleted either exon1 or exon2 or both exons of *hrp2* and *hrp3,* while in the 2016 survey we only observed parasites deleting both exons of *hrp2* and *hrp3*. This suggests that there likely have been an outbreak of dual *hrp2/3*-deleted *P. falciparum* in the Northern Red Sea zone during the 2015–2016 transmission season. Importantly, our data, combined with several recent reports of significant proportions of single exon deletion of *hrp2/3* genes in other areas^8,13^, suggest molecular assays focusing on only one of the exons in *hrp2/3* genes^14–16^ could underestimate a large proportions of *hrp2/3*-deleted parasites in some areas.

In conclusion, this study demonstrates that although lower, the prevalence of *hrp2/3* deleted parasites remains high in Eritrea 2 years after the country switched away from HRP2-based RDTs. While the prevalence is highly heterogeneous within and between different zones, HRP2-based RDTs remain unsuitable for malaria diagnosis in the country. Results also demonstrate *hrp2/3*-deleted parasites have multiple haplotypes and many are shared with or connected with parasites without gene deletions, indicating that these mutant parasites have highly likely evolved from multiple and local parasite genetic backgrounds. Therefore, although preventing spread of these mutants is important, it is likely that all areas using HRP2-based RDTs are at risk of the same phenomena. A better understanding of the factors driving selection beyond use of HRP2-based RDTs is needed. Continued surveillance on the prevalence of *hrp2/3*-deleted parasites in the affected zones in Eritrea and neighbouring countries using the WHO standard survey protocol^17^ is required to better understand the prevalence trend and to continuously inform malaria diagnosis and case management policies.

## Methods

All methods were performed in accordance with the relevant guidelines and regulations.

### Survey sites, patient recruitment and sample collection

A cross-sectional surveillance involving key malaria endemic sites in Eritrea was conducted during the transmission season 2019–2020 (Jan 2019-Jan 2020) in 11 health facilities across the 4 zones where *hrp2/3* deletions or high false negativity rate have been previously reported^2,3^ (Table 1). Suspected malaria cases seeking treatment at these facilities were tested by microscopy. Patients meeting inclusion criteria (confirmed *P. falciparum* infection by microscopy; both sexes with ages ≥ 1) were informed about the survey and invited to give consent for participation. Enrolment was consecutive until the required sample number was reached or at the end of transmission season.

### Sample collection

Eligible patients were finger pricked to produce 3–5 blood spots (~ 75–80µL each) on Whatman Protein Saver Cards. Cards were air-dried for 24 h, placed in sealed plastic bag with desiccant and stored at room temperature.

### DNA extraction and Plasmodium speciation

Genomic DNA was extracted from 3 punches of a dried blood spot (DBS) using QIAamp DNA Mini Kit and a QIAcube (QIAGEN) following the manufacturer’s instructions^3^. *Plasmodium spp* was detected using an 18S rRNA gene-based multiplex PCR assay^18^. *P. vivax* infections were confirmed by PCR using *P. vivax* specific primers alone.

### Characterization of hrp2/3 gene deletions

PCR assays were used to detect the presence of exon1 across exon2 (exon1-2) and exon2 of *hrp2* and *hrp3* genes in each sample as previously described^19^. PCR assays were also used to detect the presence of *msp1* and *msp2* genes in the same sample. As previously described, samples were classified as infected with parasites having deleted *hrp2/3* genes if they were PCR negative for exon1-2 and/or exon2 of the *hrp2* and/or *hrp3* gene, but were PCR positive for both *msp1* and *msp2* genes^20^.

### HRP2 levels

Levels of HRP2 in DBS samples were measured for all samples classified as having gene deletions (n = 311) and a randomly selected set of samples without gene deletions (n = 105) using a Quantimal Celisa Pf HRP2 Assay kit (Cellabs, Cat number: KM 810). The assay involves two steps:

1. Elution of parasite proteins from a second DBS following a published method^21^. Briefly, one DBS was punched into a 1.5 ml Eppendorf tube (3 punches), then eluted by adding 250 µl Elution Buffer (PBS/0.05% Tween 20). The tube was vortexed, spun to keep DBS submerged and followed by shaking in Thermomixer (12 °C, 350 rpm) for 12 h. During the first three hours of shaking, each tube was vortexed for 30 s, spun and returned to the Thermomixer once every hour. After shaking, the tubes were centrifuged at 13, 000 rpm for 3 min. Eluate (200 µl) was transferred to a new tube and 100 µl used for the CELISA HRP2 assay. If not immediately assayed, the eluate was stored at − 20 °C for 2 days and assayed.
2. ELISA following the manufacturer’s instructions. Briefly, 100 µl of each serial dilution of positive control, test sample, HRP positive control sample (Pf 3D7), HRP negative control sample (Pf 3BD5), normal RBC control was pipetted into individual wells. The contents were mixed by pipetting up and down 4 times. The plate was covered, placed in a humid chamber and incubated for one hour at 37 °C. The plate was then washed 4 times with wash buffer (PBS-Tween 20). Conjugate (100 µl/well) was added and the plate was incubated in a humid chamber for one hour at 37 °C. This was followed by 4 washes with wash buffer. The plate was inverted and dried on a sheet of paper. Freshly prepared Substrate Solution (TMB, 100 µl/well) was added, the plate was covered with foil and incubated for 15 min at room temperature. Stop Solution (50 µl/well) was added and results were read in a spectrophotometer (TECAN) at 450 nm wavelength.

Final absorbance OD_450nm_ for each sample was normalised with the average of OD_450nm_ from normal human blood (n = 40). ΔOD_450nm_ (OD_450nm_ sample—OD_450nm_ normal human blood) was used to represent the quantity of HRP2 in the DBS sample.

### Microsatellite analysis

Seven neutral microsatellite markers (TA1, PolyA, PfPK2, TA109, 2490, 313, and 383) were analysed for a total of 180 samples as previously published^22–24^. These included 20 samples selected from each health facility to represent local parasites with different *hrp2/3* status. Five of the seven microsatellite markers (TA1, PolyA, PfPK2, TA109 and 2490) were amplified by semi-nested PCRs^22^ while the remaining two microsatellite markers (313 and 383) by a single round PCR^23^ using published primers and PCR conditions. Sizes of fluorescent labelled PCR products were analysed on an ABI 3100 Genetic Analyzer sequencer (Applied Biosystems). The microsatellite fragments/alleles were scored manually using Peak Scanner Software version 1.0 (Applied Biosystems, https://peak-scanner-software.software.informer.com/1.0/) and a peak height > 300 relative fluorescence units (rfu) was considered as a positive peak^3^. This criteria is more stringent than that (> 200 rfu) used in earlier studies^22,25^. A laboratory line (3D7) was included in each run for size calibration.

### Haplotype construction, genetic diversity—expected heterogeneity (H_***E***_) and genetic relatedness

Seven-microsatellite-marker-haplotypes were constructed from the most dominant allele at each marker for samples with positive peaks at all seven markers. Haplotypes in relation to health facilities and gene deletion status were imported into the FSTAT software (V2.9.4) and calculated. Genetic relatedness analysis was conducted using seven-microsatellite-marker-haplotypes and PHYLOViZ online software (version 1.1) following instructions.

### Statistical analysis

Prevalence of deletions was compared between selected health facilities using Fisher’s exact test, and between zones using an Independent Samples Chi-square test in Graphpad Prism (version 9). HRP2 levels between samples of different *hrp2/3* gene status and genetic diversity between samples of different health facilities and *hrp2/3* status, were compared using Kruskal–Wallis test with Dwass–Critchlow–Fligner pairwise comparison in Jamovi (version 1.8.1). Confidence intervals for all proportions were calculated using the modified Wald method.

### Sequencing hrp2 exon2

DNA sequencing was performed for a single sample that was determined as single *hrp2*-deletion but had a detectable HRP antigen level. PCR product of *hrp2* exon2 was excised from an agarose gel, purified using a Zymoclean™ gel DNA recovery kit and sequenced using Big-dye^26^. DNA sequence was analysed and translated into protein sequence using MEGA4.02 software^27^. Amino acid repeat type was determined as previously described^26^.

### Ethical consideration

The study was approved by the Eritrean MOH Research and Ethical Committee. Patients were enrolled after providing informed consent following a detailed explanation about the investigation. For participants under 18, informed consent was obtained from a parent and/or legal guardian. All patient specimens were given a unique identifying (ID) number after collection and only this ID was used for data linkage. Laboratory analyses on *hrp2/hrp3* status, genetic diversity and relatedness conducted was approved by the Australian Defence Joint Health Command Low Risk Ethics Panel (LREP 15-004).

## Supplementary Information

Supplementary Information.

## Data Availability

Data generated from this study are included in the published article or as supplementary information. Data are also available from corresponding author on request.
