# Supplementary material for: Epidemiology of mutant Plasmodium falciparum parasites lacking histidine-rich protein 2/3 genes in Eritrea 2 years after switching from HRP2-based RDTs
Source: Sci Rep. 2021 Oct 26;11:21082. doi: 10.1038/s41598-021-00714-8 (PMC8548324; doi:10.1038/s41598-021-00714-8)
Supplement: Supplementary file 1 — Supplementary Information. [file 41598_2021_714_MOESM1_ESM.pdf]

Epidemiology of mutant *Plasmodium falciparum* parasites lacking histidine-rich protein 2/3 genes in Eritrea two years after switching from HRP2-based RDTs

Selam Mihreteab[1#], Karen Anderson[2,3], Cielo Pasay[2,3], David Smith[2,3], Michelle L. Gatton[4], Jane Cunningham[5], Araia Berhane[6], Qin Cheng[2,3#]

[1]National Malaria Control Program, Ministry of Health, Asmara, Eritrea; [2]The Australian Defence Force Malaria and Infectious Disease Institute Laboratory, QIMR Berghofer Medical Research Institute, Brisbane, Australia; [3]Drug Resistance and Diagnostics, Australian Defence Force Malaria and Infectious Disease Institute, Brisbane, Australia; [4]School of Public Health and Social Work, Queensland University of Technology, Brisbane, Australia; [5]Global Malaria Programme, World Health Organization, Geneva, Switzerland; [6]Communicable Diseases Control, Ministry of Health, Asmara, Eritrea.

[#]Corresponding authors

Selam Mihreteab, National Malaria Control Program, Ministry of Health, Asmara, Eritrea, email: smuqubay@gmail.com;

Qin Cheng, Australian Defence Force Malaria and Infectious Disease Institute, Brisbane, Australia, email: qin.cheng@defence.gov.au

Supplementary Figures

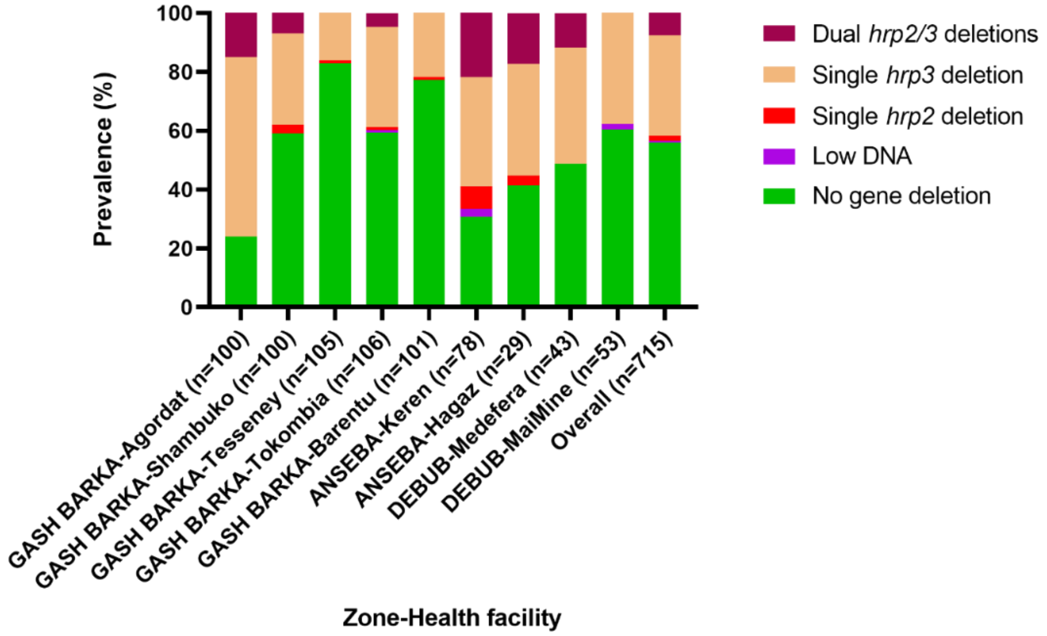

Supplementary Figure 1. Prevalence and geographical distribution of *hrp2* and *hrp3* deletions in *P. falciparum* positive samples.

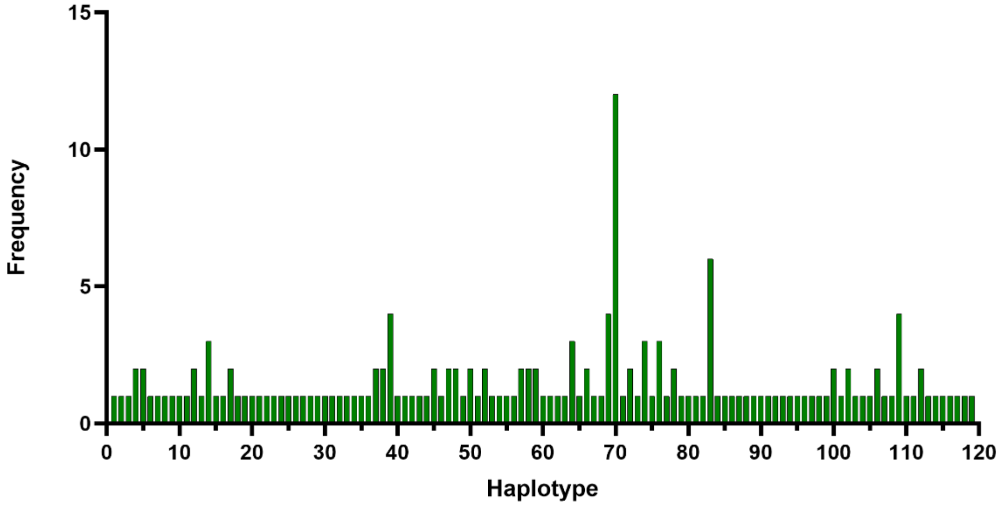

Supplementary Figure 2. Frequency of dominant haplotypes observed in 173 samples.

Supplementary Tables

Supplementary Table 1. Number of samples examined for seven microsatellite markers and their *hrp2/3* gene status.

Zone	Facility*	Number tested	Single *hrp2* deletion	Single *hrp3* deletion	Dual *hr2/3* deletion	No *hrp2/3* deletion
GASH BARKA	Agordat HP	20	0	10	5	5
	Shambuko HC	20	1	9	4	6
	Tesseney HP	20	1	11	0	8
	Tokombia HC	20	5	5	5	5
	Barentu HP	20	1	10	0	9
ANSEBA	Keren HP	20	1	8	4	7
	Hagaz HC	20	1	9	5	5
DEBUB	Mendefera HP	20	0	9	5	6
	MaiMine HC	20	0	9	0	11
	Total	180	10	80	28	62

* HP: Hospital, HC: Health Centre.
